# Occupational Exposure to Electromagnetic Fields—Different from General Public Exposure and Laboratory Studies

**DOI:** 10.3390/ijerph20166552

**Published:** 2023-08-09

**Authors:** Kjell Hansson Mild, Mats-Olof Mattsson, Peter Jeschke, Michel Israel, Mihaela Ivanova, Tsvetelina Shalamanova

**Affiliations:** 1 Department of Radiation Sciences, Radiation Physics, Umeå University, 90187 Umeå, Sweden; kjell.hansson.mild@umu.se; 2 SciProof International AB, 83158 Östersund, Sweden; 3 Institute of Advanced Studies, Strömstad Academy, 45280 Strömstad, Sweden; 4 Federal Institute for Occupational Safety and Health, 44194 Dortmund, Germany; peter.jeschke@vodafone.com; 5 National Centre of Public Health and Analyses, 1431 Sofia, Bulgaria; michelisrael@abv.bg (M.I.); mihaela_1970@abv.bg (M.I.); ts.shalamanova@gmail.com (T.S.)

**Keywords:** electromagnetic field, biological effects, health surveillance, workers, general population

## Abstract

The designs of in vivo, in vitro and in silico studies do not adequately reflect the characteristics of long-term occupational EMF exposure; the higher exposure levels permitted for employees are nevertheless extrapolated on this basis. Epidemiological studies consider occupational exposure only in a very general way, if at all. There is a lack of detailed descriptive data on long-term occupational exposure over the duration of the working life. Most studies reflect exposure characteristics of the general population, exposures which are long-term, but at a comparably low level. Occupational exposure is often intermittent with high peak power followed by periods with no exposure. Furthermore, the EU EMF-Directive 2013/35/EU states a demand for occupational health surveillance, the outcome of which would be of great help to epidemiologists studying the health effects of EMF exposure. This paper thus aims to outline and specify differences between public and occupational exposure and to increase the understanding of specific aspects of occupational exposure which are important for long-term health considerations. This could lead to a future protection concept against possible hazards based on adequate descriptions of long-term exposures and also include supplementary descriptive features such as a “reset time” of biological systems and accurate dose quantities.

## 1. Introduction

ICNIRP’s and IEEE’s safety concepts [1,2,3] are widely acknowledged, providing the basis of electromagnetic field (EMF) legislation in many countries worldwide. The mechanisms of action for thermal effects at radiofrequency EMF and stimulation effects at low-frequency EMF are well understood. In a recent paper Barnes and Freeman discuss the problem of setting safety guidelines based on low-level EMF [4]. They suggest that some data support that biological systems can respond to weak RF field exposure without significant changes in temperature. In their summary, they say that data indicate that low-level RF power can lead to cumulative effects generally not seen for short-term exposure.

However, the designs of in vivo, in vitro and in silico studies do not adequately reflect the characteristics of long-term occupational EMF exposure; the higher exposure levels permitted for employees are nevertheless extrapolated on this basis. Moreover, in epidemiological studies, occupational exposure is only considered in a very general way, if at all. There is a lack of detailed descriptive data on long-term occupational exposure over the duration of the working life.

Along with the latest publications, [1,2,3], a reduction of safety factors between avoidable health effects (threshold level) and tolerable biological effects (basic restrictions) sparked discussions regarding the comprehensiveness and diversity of the scientific bases used by guideline setters [5,6,7]. The underlying science is primarily composed of results from animal studies, computer simulations and epidemiological studies [8]. So far so good, if it were not to be mostly studies reflecting the exposure characteristics of the general population. Focusing mostly on studies with an emphasis on general public exposure is problematic if safety guidelines for occupational settings are extrapolated solely from these. This paper explains in what way exposure characteristics for the general public and occupational exposure differ, argues why a reduction of safety factors matters for safety at workplaces and proposes measures for improving the quality of statistical data, i.e., correlations between exposure and the possible occurrence of health effects.

## 2. Occupational and General Public Exposure Characteristics

To start with, we introduce exposure scenarios of the general public (Section 2.1) in contrast to workplace scenarios with relevant EMF exposure (Section 2.2). Based on that, the differences in both exposure scenarios are evaluated and characteristics described (Section 2.3).

### 2.1. General Public Exposure

The main EMF sources contributing to the exposure of the general public are listed below in order of increasing frequency:

- Low-frequency EMF: public transport, like trams and trains (engine and heating); electrical power supplies, like overland power lines, transformer stations, cables and wall sockets; household appliances, like power tools (drills, grinders), irons, kitchen aids, electrical razors; personal mobility, like battery cables in older cars, or cable-based or wireless charging of electrical vehicles; induction cooking; nearfield wireless power transfer; antitheft devices and electronic article surveillance.
- Radiofrequency EMF: RFID and security devices; radio and television broadcasting; smartphones, smart gadgets and devices, outdoor/indoor base stations, Bluetooth, and Wi-Fi routers; autonomous vehicles with distance radars and car2car communication.

In general, low-frequency magnetic field exposure of the general public with some magnitude often only reaches the immediate surroundings, when being operated. 

**Figure 1:**
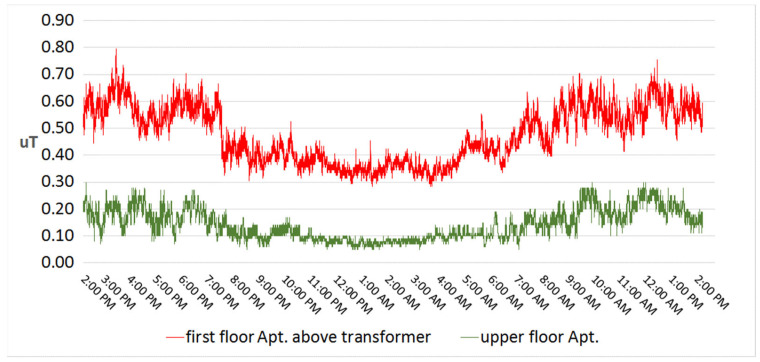
Magnetic field 24 h dosimetry (with time of the day (h) on the *x*-axis and magnetic flux density (µT) on the *y*-axis) with EmdexLite data logger on the first floor above an inbuilt transformer station and on the upper floor in same building. (Figure modified from [9]). The measurement results show clear differences between the magnetic field values measured in the floor directly above (in red) the transformer room and on other floors (in green).

Long-term low-level exposure can be found at premises near power lines or in an apartment situated above an inbuilt transformer station (Figure 1) [9]. 

In such apartments, the magnetic field is well below the reference levels according to the Council Recommendation on EMF 1999/519/EC [10] but exceeds the yearly average value of 0.4 µT considered as a precautionary cut-off point for childhood leukemia [11]. Investigating the underlying interaction mechanism leading towards a risk for childhood leukemia is a current research challenge [12].

Due to the nature of motors of handheld power tools, which are comparable to a coil, the effective distance of the magnetic field is comparably small, causing only the local exposure of the hands and perhaps the chest when being operated. It relates with the inverse “cube” law: a doubling in distance results in an approximate eightfold decrease in intensity.

Among modern means of telecommunication, sources emitting radiofrequency EMF expose different parts of the human body due to their pattern of use. Broadcasting sites and base stations contribute to whole-body far-field exposure, whereas smartphones and smart gadgets only cause the local exposure of the head and hands.

A report of the Scientific Committee on Emerging and Newly Identified Health Risks for the European Commission (SCENIHR, renamed SCHEER since 2016) of 2015 [12] presents a summary of studies on electromagnetic exposure around radio and television transmitters, showing that, despite the lower emitted power after digitization, a statistically significant increase in the average EMF exposure in the frequency range used for television broadcasts is reported. Nevertheless, compared to [5], the measured values of the electric field strengths and the power densities around radio and television stations are well below the reference levels.

In the European single market, exposure levels in the general public are well regulated, e.g., by harmonized product standards referencing [13]. Compliance is under surveillance by competent authorities. Overall, the EMF exposure of the general public is reasonably low, compared to permissible values [14]. Outside of Europe, monitoring activities of general public exposure are few and primarily refer to radiofrequency exposures from base stations. Examples include the studies by [15,16]. The first of these ones collected data from surveys of radio base stations from 23 countries from five continents. The data were from the year 2000 onwards and included 173,000 data points. In summary, ground-level radio frequency signals were well below recognized exposure guidelines. Similar data were seen in the second of these studies, where 260,000 measurements from seven African countries were analyzed. The mean levels of exposure in this continent were comparable to levels found in Europe, Asia, Oceania and North America. A recent Japanese study [17] measured E-field levels in Tokyo and surrounding prefectures during the period 2021–2022. They found that mobile phone base stations dominated over FM/TV frequency bands and that there was a positive correlation between E-field strengths and population density. The measured levels were far below the national radiation protection guidelines. Another recent study also found a correlation between population and building density and measured E-fields, in the Beijing urban region [18]. The highest measured levels in the frequency range from 100 kHz to 6.5 GHz were above 4 V/m, although most measurements were below 1.5 V/m. 

The ICNIRP guidelines from [1,3,19] are still not implemented in European general public legislation, and only partly incorporated into specific requirements regarding workers’ EMF exposure management. Furthermore, some European countries, such as Belgium, Bulgaria, Germany, Italy and others, have defined more restrictive national legislation for the protection of public health from exposure to EMF. Hence, exposure scenarios where measured EMF values could reach the nation-specific stricter limit values for the general public might occur.

### 2.2. Occupational Exposure

**Figure 2:**
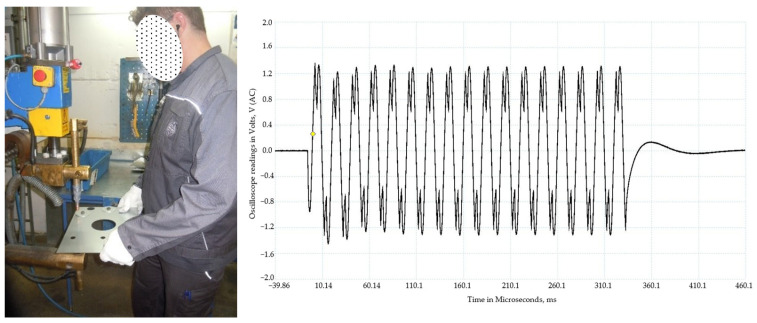
Operator of a spot-welding machine, operating with tens of kA in current. Each weld typically lasts for ten or more periods of the 50 Hz current. The oscilloscope picture shows the recording of the magnetic field, and the EU directive action levels are exceeded inside a distance of 0.4 m to the electrode (Hansson Mild, personal communication).

Occupational exposure to electromagnetic fields seldom occurs with a constant intensity for hours but it is often intermittent with a high peak power followed by periods with no exposure [20]. This can be exemplified by the exposure to an extremely-low-frequency magnetic field (ELF-MF) for an operator of a spot-welding machine (Figure 2). A typical weld is about 10–15 periods of 50 Hz current, where the current is of the order of tens of kA, leading to an exposure of about a few milliTesla (mT) on the part of the body closest to the electrode. This can then be repeated one or two more times per minute followed by a longer period with a change in the material to be welded. The operator often uses the same machine the whole working day, or in some cases half of their working hours are spent on another task without this high exposure.

**Figure 3:**
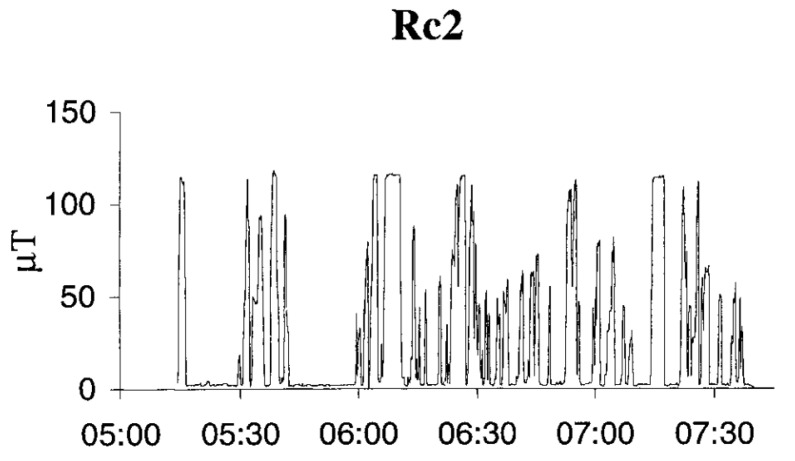
Magnetic field recordings when the driver on an RC2 freight train engine kept a logger (EmdexLite) in the breast pocket of their jacket. The diagram shows that the logger was sometimes saturated, giving the maximal reading of 120 µT. The curve also illustrates the intermittent character of the exposure. Time of measurement is depicted on the *x*-axis, with magnetic flux density (µT) on the *y*-axis (Hansson Mild, personal communication).

Another example of intermittent exposure comes from engine drivers of electric trains. Here, the exposure to the ELF-MF is high when starting and stopping the train, but also intermittent during the journey. The daily average is in the order of 5 to 15 µT, depending on the type of engine (Figure 3; see further in [21]). Exposure of workers to electric and magnetic fields in the production of electricity could be characterized by the type of energetic system, professional group and particular duties. As the electric field (EF) strength depends on the voltage, values up to tens of kV/m can be found in 400 kV distribution systems. The ELF-MF field strength depends on current consumption and the maximal values of the order of tens of µT are found in internal and external distribution systems. Even higher values can occasionally be found, e.g., at hydropower stations where there are areas around turbines and generators with ELF-MF values around 0.1 mT. The daily exposure scenario for this occupational group consists of periods of the day with particular tasks performed in areas with background levels of electric and magnetic fields in command halls, and other tasks performed in areas with very high field levels [22].

**Figure 4:**
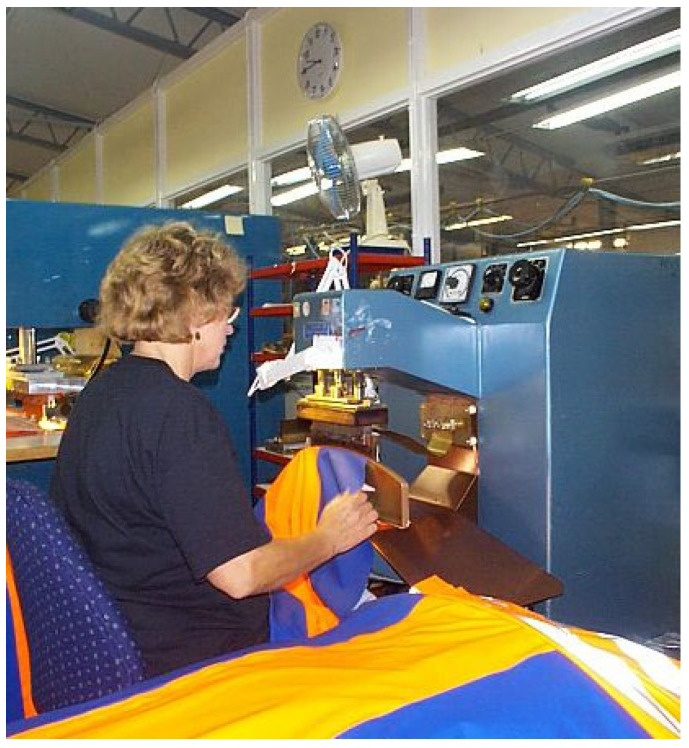
Operator of a plastic welding machine. The exposure of the hands can be quite substantial. The body also gets a rather high exposure. The weld typically lasts for some to a few seconds with a repletion time of some tens of seconds (Hansson Mild, personal communication).

When it comes to exposure from radiofrequency fields (RF), it is pretty much the same as for the ELF-MF range. The operators of different machines are sitting or standing in front of, e.g., an RF plastic welding sealer and operate it in an intermittent way dependent on the product being manufactured (Figure 4). In the ready-made clothing industry, the operator is working with their hands very close to the electrodes with a resulting high local exposure of the hands. Some machines also give rise to rather high whole-body exposure, especially in the head and knees which can be close to the electrodes. Usually, the welding time is of the order of 2–4 s, with 30–60 s in between the next weld.

Particular attention should be paid to occupational exposure to complex/multifrequency fields, such as magnetic resonance imaging (MRI) equipment emitting in several frequency ranges, characterized by an inhomogeneous distribution of EMF: large gradients of a magnetic field occurring in the shielded/procedure chamber. Medical personnel could be exposed during assistance of children or a patient in a severe condition, and the injection of a contrast agent or anesthesia in an MRI chamber (see further in [23]).

Another medical occupation that deserves special consideration is physiotherapists, where the exposure is to different sources emitting in several frequency ranges. The exposure is inhomogeneous, with different durations depending on the source and procedures, like in diathermy. Data of measurements and exposure assessment show high exposure to the personnel, especially in the RF range [24].

In the EU Directive 2013/35/EU on occupational exposure to EMF [25] article 8, it states the demand for health surveillance: “With the objective of the prevention and the early diagnosis of any adverse health effects due to exposure to electromagnetic fields, appropriate health surveillance shall be carried out in accordance with Article 14 of Directive 89/391/EEC.” The text further states: “If any undesired or unexpected health effect is reported by a worker…. the employer shall ensure that appropriate medical examinations or individual health surveillance is provided to the worker(s) concerned….”. Unfortunately, so far, this seems to not have been carried out to any large extent in EU countries, except for workers at particular risk. The outcome of such health surveillance would be of great help to epidemiologists studying the health effects of EMF exposure.

Examples of such health surveillance can be found among the former East European countries. There are specific requirements set in some national legislations concerning mandatory health surveillance according to the risk factor and professional group [26]. These include mandatory pre-employment and periodical medical examinations according to indications/complaints and specific laboratory tests as well. Many examples can be found in the proceedings from a workshop on clinical and physiological investigations of occupational EMF-exposed persons (see further in [27]). Among the studies, the following can be mentioned: dysregulation of the autonomic control of cardiac function and a shift of the diurnal rhythms of blood pressure in workers exposed to RF-EMF; a suppression of immunological reactivity; stress hormone excretion rates in broadcast stations; physiotherapy and broadcast stations; and cardiovascular risk morbidity with temporary disability among electric substation workers (see [22,28,29,30]).

These types of studies have earlier been mentioned in the books by [31,32,33] but they were never followed up to any larger extent in the literature in the West. However, such investigations would be very helpful in the continuing work to establish safety guidelines for occupational exposure.

There is a lack of data in the scientific literature from studies of the human health effects from static magnetic fields up to 8 T from MRI devices, including long-term effects. The few exceptions regarding the effects of long-term exposure include studies of hypertension development among workers at an MRI device manufacturing facility [34].

A few occasional studies have investigated perceived symptoms related to static magnetic field exposures among MRI workers, as discussed in further detail in [35]. The majority have focused on exposures of ≤3T, since devices at these flux densities are the most commonly used for diagnostic purposes. The exposure is mainly associated with transient sensory effects such as vertigo, nausea and magnetophosphenes as well as cognitive effects, such as concentration problems (see [35] for further details).

The transition between being a worker and becoming part of the general public can sometimes change rapidly. The EU Directive [25] states that an individual risk assessment should be carried out for workers who have declared the use of active or passive implanted medical devices, such as cardiac pacemakers, or the use of medical devices worn on the body, such as insulin pumps, or in respect of pregnant workers who have informed their employer of their new health status. In this case, the occupational limits become those for the general public; see further in [36]. Often, when an exposure assessment is being carried out at workplaces with possible high exposure, it is necessary to cover both the occupational and general public limits and inform the workers of the outcome, so that they are aware of that they have an obligation to inform the employer of any change in the status that would require a new risk assessment.

Experimental studies that specifically or indirectly address occupational exposures are few. One example of an attempt to address occupational situations has been published by [37], who exposed male Kunming mice to a 0.50 mT 50 Hz magnetic field for 8 h/day, 5 days/week, for 60 days, using the ICNIRP guidelines’ reference levels for occupational exposure as a guide to the exposure conditions. The study focused on the spleen and splenic lymphocytes and found a decreased bodyweight at days 20 and 30 of exposure, but no other effects from this exposure. Primary cultures of splenic lymphocytes were also used in vitro, where exposures were to this 50 Hz field (at 0, 0.25, 0.50, and 1.0 mT) for 6 h as an acute exposure paradigm. The investigated endpoints, mRNA expression of a battery of cytokines, did not show any effects of the exposure. Other examples of at least occupationally relevant exposures include a study from [38] on porcine platelets in vitro (exposure mimicking E-field from LCD computer monitors), with a focus on malondialdehyde concentration, and from [39] on adult male mice exposed to 50 Hz 2 h/day for 60 days, regarding inflammatory cytokines. These and other studies show contradictory results and are furthermore not especially relevant for either any occupational exposure or any specific health concern in the occupational setting.

### 2.3. Evaluation of Exposure Characteristics

Based on the descriptions in the previous sections, it becomes obvious that occupational exposure settings differ significantly from those of the general public. Occupational exposure is distinguished by:

- Higher magnitudes of dominant field components;
- The frequency ranges used;
- Longer exposure durations;
- Parallel exposure from neighboring workplaces or machinery;
- Complex signal shapes instead of sinusoidal wave forms;
- Smaller distances between EMF source and operator with mainly near-field exposure, especially in the low frequency range;
- High levels of permitted peak values of pulsed exposures at RF-EMF instead of time-averaged values for continuous exposure;
- Intermittency, for example based on the operating procedures of industrial or production processes.

In addition to the above listed characteristics, there is a difference in the level of permitted exposure according to exposure guidelines [1,3,40]. This is reasonable, because occupationally exposed workers are trained in operating the EMF sources at the workplace, and, hence, are considered to be exposed under controlled conditions in relation to their working tasks and are trained to employ harm-mitigation measures. Furthermore, it is assumed that healthy workers, ranging from 16 to 65 years of age, also have the cognitive capacity to employ those measures.

This justifies a higher level of permitted exposure for workers. In general, occupational exposure is permitted to be two to five times higher than general public exposure [1,3,25], based on exposure limit values/basic restrictions. To compare those “gross” levels of permitted exposures, it is important to look into the details. Different time windows and exposure durations need to be accounted for when comparing exposure levels of both general public and workers:

- General public: 24 h × 7 d = 168 h of off-duty “leisure” time per week,
- Occupationally exposed workers: 8 h × 5 d = 40 h of on-duty working time in combination with 16 h × 5 d + 24 h × 2 d = 128 h of off-duty “leisure” time per week.

In the next step, weekly time frames are used to calculate a dose-like measure to illustrate the net difference in exposure levels, assuming workers are exposed at the maximally permitted level during working hours (own calculations based on [1,3])

- Low frequency range to prevent
  - central nerve stimulation, net factor of ($\frac{\left(5\frac{occexp}{genpub}\;\cdot \;8\;hrs\;\cdot \;5\;d\right)+\left(1\frac{occexp}{genpub}\;\cdot \;16\;hrs\;\cdot \;5\;d\right)+\left(1\frac{occexp}{genpub}\;\cdot \;24\;hrs\;\cdot \;2\;d\right)}{\left(1\frac{occexp}{genpub}\;\cdot \;24\;hrs\;\cdot \;7\;d\right)}=\frac{328}{168}=1.95$ net per week (gross relation occup vs. gen pub of 5)) (gross factor was 5×).
  - peripheral nerve stimulation, net factor of ($\frac{\left(2\;\frac{occexp}{genpub}\;\cdot \;8\;hrs\;\cdot \;5\;d\right)+\left(1\frac{occexp}{genpub}\;\cdot \;16\;hrs\;\cdot \;5\;d\right)+\left(1\frac{occexp}{genpub}\;\cdot \;24\;hrs\;\cdot \;2\;d\right)}{\left(1\frac{occexp}{genpub}\;\cdot \;24\;hrs\;\cdot \;7\;d\right)}=\frac{208}{168}=1.23$ net per week (gross relation occup vs. gen pub of 2)) (gross factor was 2×).
- Radio frequency range: whole body and local exposure, net factor of ($\frac{\left(5\frac{occexp}{genpub}\;\cdot \;8\;hrs\;\cdot \;5\;d\right)+\left(1\frac{occexp}{genpub}\;\cdot \;16\;hrs\;\cdot \;5\;d\right)+\left(1\frac{occexp}{genpub}\;\cdot \;24\;hrs\;\cdot \;2\;d\right)}{\left(1\frac{occexp}{genpub}\;\cdot \;24\;hrs\;\cdot \;7\;d\right)}=\frac{328}{168}=1.95$ net per week (gross relation occup vs. gen pub of 5)) (gross factor was 5×).

The numbers show that workers are higher exposed by a factor of up to 1.95 per week. Adjusted for holidays and sick leave (220 working days per annum), workers are more highly exposed, approximately, by a net factor of ($\frac{\left(5\;\frac{occexp\;}{genpub}\;\cdot 8\;hrs\;\cdot \;220\;d\right)+\left(1\frac{occexp}{genpub}\;\cdot \;16\;hrs\;\cdot \;220\;d\right)+\left(1\frac{occexp}{genpub}\;\cdot \;24\;hrs\;\cdot \;145\;d\right)}{\left(1\frac{occexp}{genpub}\;\cdot \;24\;hrs\;\cdot \;365\;d\right)}=\frac{15800}{8760}=1.8$0 per week (gross relation occup vs. gen pub of 5)) per annum for central nerve stimulation and for thermal effects at RF-EMF, as well as a net factor of ($\frac{\left(2\;\frac{occexp}{genpub}\;\cdot \;8\;hrs\;\cdot \;220\;d\right)+\left(1\frac{occexp}{genpub}\;\cdot \;16\;hrs\;\cdot \;220\;d\right)+\left(1\frac{occexp}{genpub}\;\cdot \;24\;hrs\;\cdot \;145\;d\right)}{\left(1\frac{occexp}{genpub}\;\cdot \;24\;hrs\;\cdot \;365\;d\right)}=\frac{10520}{8760}=1.2$0 per week (gross relation occup vs. gen pub of 2)) per annum for peripheral nerve stimulation, as the temporal effect of leisure off-duty time vs. duty time reduces the dose-like annual measure.

However, the influence of exceeding maximal exposure levels for short time frames during a working procedure cannot be addressed with such dose-like measures. Hence, in the next step, we take a closer look into the influence of the actual working procedure. To recapture, during working hours, permitted exposures are two to five times higher for workers as for the general public with thresholds to be exhausted [41,42,43]. Being exposed so closely to maximum permissible action levels, it becomes important to numerically assess EMF exposures against exposure limit values as one means of mitigation.

For low-frequency central and peripheral nerve stimulation, the internal electrical field strength *E_i_* can be calculated based on the induction law:

curl E=−∂B/∂t

Applying this to a circular loop with a radius r and incoming perpendicular magnetic field, the equation can be simplified as follows, where *f* is the frequency:

$$E_{i}\left(r\right)=-\pi rf\mu_{0}H=-\pi rfB$$

Assuming an exposure at maximal magnetic flux of *B* = 100 µT (based on [25], table B.2, for frequencies between 3 kHz and 10 MHz) and trunk exposure with *r* = 0.6 m at *f* = 69 kHz, it results in:

$$\left|E_{i}\left(r\right)\right|=\pi rfB=3.14\cdot 0.60\;m\cdot 69\;\mathrm{kHz}\cdot 100\;µT=13\;V/m$$

The exposure is compliant with the EMF-Directive table A3 [25]:

$$ELV_{E_{i}}=3.8\cdot 10^{-4}\cdot f=3.8\cdot 10^{-4}\cdot 69\;\mathrm{kHz}=26\;V/m$$

resulting in an exposure index of 0.5.

Based on the numerical SAR assessment in [5], with *f* = 2–6 GHz and *S_inc_* = 50 W/m^2^, further evaluation provided insight that in every body height category ranging from 150 to 200 cm, a body weight of below approximately *m* = 66.56 ± 1.01 kg exceeded the exposure limit values of whole-body SAR = 0.4 W/kg. This result makes it obvious that even workers with body mass indices beyond 25, ranging from normal weight to pre-obesity, and smaller statutes of around 160 cm, are prone to be overexposed at the workplace.

## 3. Physical Considerations

In the previous section, two aspects were shown: (1) for occupational exposure scenarios, real exposures are higher than those for the general public; and (2) the need to assess exposure limit values in specific scenarios in order prevent inadmissible exposures. How to interpret these two aspects physically, as well as biologically, is the subject of the following two sections.

### 3.1. “Dose” in EM Research

Since the interaction mechanism(s) are not well understood for weak fields and nonthermal effects, the concept’s exposure and dose are not clearly defined in EMF research. There is also a need to look at different dose concepts regarding different symptoms and diseases (see further in [44] for an extensive discussion). For ELF-MF, several possible exposure characteristics have been discussed, for instance, intensity (strength) given as RMS or peak value; duration of exposure; single versus repetitive exposures or intermittent exposures; transients; frequency content of the signal; field orientation; and a combination of ELF-MF and static field.

Most epidemiological studies of health effects have, as a measure of dose, used various forms of mean values of exposure, such as the time average of the magnetic field during a working day or a 24 h day. Others have used the highest value found in a day as a measure. Only a few studies have attempted to create a dose in the form of an integration of exposure over time, such as in the form of µT hours. We do not know, however, if this is the correct dimension; is an exposure to 20 μT for 0.5 h equivalent to 0.5 μT for 20 h? Is the effect linearly dependent on the “dose” or is it, as in many biological contexts, a nonlinear relationship? This is valid for acute/deterministic effects in photobiology (Bunsen–Roscoe law of reciprocity), but it considers high energy radiation where the dose–response relationship is well known.

Are various frequencies—basic tones and harmonics—to be included when assessing the weighted average of a dose? Before these fundamental questions of what constitutes “dose” in these contexts are better understood, our risk assessment will be arbitrary when it comes to long-term exposures below applicable limits for established effects or suggested long-term effects.

Additional “dose” parameters were set as limit values in some Eastern European countries in the past [45,46]. These parameters are the energetic loading of the organism expressed as E^2^T (V/m)^2^h, H^2^T (A/m)^2^h and ST (Wh/m^2^) and set for different frequency ranges corresponding to the considered health effects. The assessment is based on the time weighted average of the field parameters, which reflect the real time of exposure to different field levels. Such an approach gives the opportunity to assess the energetic load of the organism and to calculate a permissible time duration of exposure according to the working shift/working cycle.

Depending on the type of disease studied, the exposure assessment in epidemiological studies needs to be very different. For instance, effects depending on only short-term exposures are different from diseases with long latencies such as cancer and Alzheimer’s.

For comparison, in ionizing radiation, there is a distinction between stochastic (linear non-threshold) and deterministic effects. The question of intermittent exposure also leaves many unanswered aspects, like the spacing of the repeated exposure, and if there is an effect of that exposure, what is the biological reset time, i.e., when is the system fully recuperated?

Critical questions that need to answered can be, for example (from [44]):

- For how long did the exposure last, and how often was it repeated?
- Is there a connection between high and low exposure levels, respectively, and biological and/or health-relevant effects?
- Is there any monotonous increase in response to increasing exposure levels, such as a classic dose–response relationship?
- What kind of data are there regarding short-term exposure and biological effects?
- What is the connection between long-lasting exposure and effects?
- Are there any data on intermittent exposure?
- Is it reasonable to expect a specific biological response after a certain exposure time?
- Is there any biological “reset time” among reported effects; how much time must pass before the investigated end point is back to initial levels?

### 3.2. Safety Guidelines and Time Average

The safety guidelines for human exposure to microwave and RF-EMF have, since the first versions, been aimed at short-term exposure and controlling whole-body temperatures from increasing above 1 °C or local tissue temperatures to 5 °C. Schwan suggested a limit at 100 W/m^2^ of incident power density in 0.1 h as a safety guideline in 1966 (see further in [47]). This time limit has then remained the same but transformed to the more precise 6 min time average, and for whole-body exposure, the time was set at 30 min [1,2], which is considered to be the interval needed to reach a steady-state temperature. However, the human body thermal constant is not known to the precision that these numbers imply: 6 ± 0.5 or 30 ± 0.5 min. These values should rather have been expressed as 0.1 and 0.5 h (0.1 ± 0.05 and 0.5±0.05 h) in view of the uncertainty in the thermal constant. But the main point is that only short-term exposure is considered, and in the occupational setting, the exposure can be stretched out over most of the 8 h working day. How our body would react to either a prolonged increase in body temperature over an 8 h working day is not known, nor is it known how a recurrent intermittent temperature increase would be dealt with over a whole working life. Perhaps lessons can be learned from an animal study which examined the long-term carcinogenic effects of RF-EMF [48]. This study, the so-called “NTP study” (on rats and mice), investigated the effects of levels of exposure at the maximum permissible value for the human head when using mobile phones (1.6 W/kg and 2 W/kg) and also at levels above this value (up to 6 W/kg). A key finding of the study was an increase in the number of malignant heart schwannomas in male but not in female rats. Among many comments regarding the study, [49] argued that the study left unanswered questions as to the effects of exposure to RF-EMF on body temperature. Calculations by these authors suggest that the temperature fluctuations caused by RF-EMF exposure in older (heavier) animals were underestimated because the pilot study had used temperature measurements in younger (lighter) animals. Accordingly, this underestimation increased with the age of the animals because of their larger body mass, and the temperature fluctuations increased as the animals grew (by ≥ 1.4 K in older male rats). It is possible that this may cause chronic thermoregulatory stress, which may explain some of the findings of the NTP study.

## 4. Biological Considerations

Experimental studies on different biological systems strive to elucidate interaction mechanisms, to identify health hazards, and to characterize potential risks, e.g., by performing dose–response studies. Two categories of experimental systems dominate the studies investigating the possible health risks from EMF exposures. Studies performed on animals (in vivo studies) typically employ rodents, whereas studies on cells either are conducted ex vivo or in vitro (i.e., cells in culture). Irrespective of the experimental model, study outcomes may have relevance for health concerns for both the general public and for the occupational situation.

Obviously, studies on animals provide information which is more similar to the real-life consequences of human exposures, although extrapolation from animal studies to humans have limitations. Such limitations have their origin in both the biological differences between species, but also in the (lack of) complexity that the laboratory environment offers compared to the environment where “real” exposures occur [50]. Studies on cells do not translate directly to real-life human exposure but can offer valuable insights into what kind of processes that are influenced by an agent, and about the mechanisms that underpin a specific pathological condition.

However, results from experimental biological studies, irrespective of the biological model, need to be interpreted cautiously, since both experimental conditions as well as outcomes may be of limited relevance for a real-life situation. Due to practical reasons, the experimental situation is often streamlined as much as possible to allow for the set-up and implementation of the experiments. This may at the same time compromise the biological relevance of the study and deviate to a substantial degree from the relevant exposure situation, especially for occupational exposures that, as described above, can be very complex. The complexity of the exposure situation can be illustrated by the absolute minimum of characteristics that are needed for a description. These include the frequency, the possible modulations, and different waveforms or pulses of the signal; the duration and continuity/intermittence of exposure; and the field strength (magnetic flux density/specific absorption rate—SAR/power density, depending on the frequency) of the electromagnetic field.

There are a number of biological caveats that can influence the interpretation of experiments. Among these are the time scales and time constants of biological processes and events. If these are not considered and “matched” to the experimental setting, a specific investigation may produce results that are irrelevant for the specific question which is asked, despite the fact that the technical aspects of the study are state-of-art. One such consideration regards circadian rhythms (see [50,51] for overviews and references to further relevant articles). Naturally, circadian rhythms are not the only factors that, for various reasons, can have an influence on the specific response of a biological system, al-though they are highly relevant for occupational health issues. Animal experiments involving EMF exposures are typically long-term (from days to months to years) and performed under conditions where normal rhythmicity (i.e., 12 h light:12 h dark) is present. However, many epidemiological studies show that disturbed circadian rhythmicity, present in many occupational situations, is in itself a risk factor for pathological conditions [51]. EMF exposures under such conditions do occur and would be of high relevance for experimental studies, e.g., those investigating co-carcinogenicity where a factor is promoting the effect of the carcinogenic agent. Furthermore, [52] has shown that few experimental animal studies correctly describe temporal details when, during the daily rhythm, experimental actions take place. Since experimental animals are mostly rodents, and nocturnal, it makes extrapolation to diurnal humans even more difficult.

Many biological processes on the cellular and molecular levels in eukaryotic cells have time constants that need to be considered for both experimental design and for the interpretation of results. Important ones include the time needed by a cell for macromolecule synthesis (DNA replication, RNA transcription, protein translation), for intracellular transport, cell motility, cell duplication, ion channel opening and inactivation, cellular lifespan, etc. (see, e.g., [53,54]). Also, possible refractory periods (“reset times”) for the action of external agents exist and need to be considered. Such time constants vary substantially and range from milliseconds to days, months and years. It is thus necessary to understand the time scales when designing exposure scenarios, so that an investigated endpoint is relevant to investigate under the chosen conditions [55]. An interesting illustration of this is provided in a study from [55], who investigated murine hematopoietic cells exposed to 50 Hz magnetic fields and the effects on DNA damage and repair, and the expression of circadian rhythm genes. Core circadian rhythm genes were not affected by exposures (15 min, 2 h, 12 h, 24 h) but some associated genes changed their expression after 12 and 24 h of exposure, and DNA repair capacity was decreased after 24 h of exposure. This illustrates nicely that a full cycle of cell duplication is needed to respond to the exposure in this case, and that shorter exposures would never pick up such consequences of magnetic field exposure.

Another important aspect refers to the choice of studied biological effects, “endpoints”, in experimental studies. If the studies are supposed to provide relevant information that can be translated into clinical practice and policy, they must employ appropriate endpoints [56]. The connection between observed changes in basic biological processes and a particular adverse health condition may be tenuous at best. As an example, a noted increase in reactive oxygen species is sometimes overinterpreted to imply a promotion of a cancerous process, or a degenerative condition.

Still other caveats in experimental studies are related to more technical issues. Exposure in a laboratory setting is often oversimplified, consisting of exposure to a certain frequency at a given magnitude for a specified time, without considering that exposures in real-life scenarios are irregular, both regarding frequency components (negligence of co-exposures and combined exposures), duration and field intensity. If, for example, intermittent exposures (on-off cycles) are used, they are often arbitrarily chosen, and do not consider either biological time scales or exposure patterns.

## 5. Knowledge Gaps, Challenges and Research Recommendations

There are several challenges that need to be overcome to improve the understanding of the possible health effects of occupational EMF exposure. Among these challenges is the lack of suitable experimental studies. Furthermore, due to a lack of proper surveillance programs, it is difficult to establish if there is any clear link between a worker’s health conditions and occupational exposure.

Relevant experimental studies have to be based on realistic exposure scenarios, of which there are many kinds. Experimental studies also have to address relevant health-connected endpoints and consider the temporal aspects of biological processes. The question about reciprocity is also an unanswered question: is, for example, a 1 min mobile phone call 10 times equal to a 10 min call once? In this context, the question of recuperation times between the calls/exposure, transient effects, adaptation mechanisms, etc., needs to be answered. Regarding thermal effects, how about the temperature increases close to maximal permissible temperature rise by intermittent exposures?

To adequately describe occupational long-term exposure and to protect against possible hazards, supplementary descriptive features, such as “repair constants” of biological systems or dose quantities, may have to be considered by a future protection concept.

Studies at population level of retired workers with a resilient link between a continuously developing health status and working life exposure are missing, too.

## 6. Conclusions

Knowledge about the possible effects of EMF on occupational safety and health is primarily obtained from research on general public exposure conditions. Thus, experts on occupational safety and health in competent authorities may from time to time face relevant questions where answers are not based on a satisfactory knowledge fundament.

The purpose of writing this article has been to provide arguments why science needs study designs which differentiate clearly between both the general public and the workers’ domain. To facilitate such a process, exposure patterns of both domains were introduced and evaluated, followed by physical and biological explanations referring to time-related exposure quantities for non-ionizing radiation protection. There are many observed, and potentially relevant, biological effects noted after exposures to both electric, magnetic and electromagnetic fields, where findings related to oxidative stress mechanisms have received particular attention [57,58]. Such findings have raised the awareness for time-related constants on the molecular and cellular levels of biological systems. However, it remains to be seen if biological effects on such a “micro” level will result in manifested health effects on a “macro” level organism. To monitor those health effects, workers need to be subjected to continuous health surveillance. And another question must be considered: which adequate genetic, blood, or, e.g., MRI or other diagnostic markers are most appropriate to be included in such a surveillance?

To tackle the above research aspects and questions as well as to promote further research into EMF at the workplace, researchers need sufficient funding and international collaboration. What is required is a new research framework with an emphasis on occupational EMF exposure at the scale of the Horizon Europe projects to spark basic and applied research with a broad focus on frequency ranges and EMF sources, exposure characteristics, as well as interaction and repair mechanisms on micro-, meso-, and macro-levels. Let us get started. 

## Data Availability

Not applicable.
